# Bone Marrow Mesenchymal Stem-Cell-Derived Exosomes Ameliorate Deoxynivalenol-Induced Mice Liver Damage

**DOI:** 10.3390/antiox12030588

**Published:** 2023-02-27

**Authors:** Zitong Meng, Yuxiao Liao, Zhao Peng, Xiaolei Zhou, Huanhuan Zhou, Andreas K. Nüssler, Liegang Liu, Wei Yang

**Affiliations:** 1 Hubei Key Laboratory of Food Nutrition and Safety, Department of Nutrition and Food Hygiene, Tongji Medical College, Huazhong University of Science and Technology, Hangkong Road 13, Wuhan 430030, China; 2 MOE Key Lab of Environment and Health, Department of Nutrition and Food Hygiene, School of Public Health, Tongji Medical College, Huazhong University of Science and Technology, Hangkong Road 13, Wuhan 430030, China; 3 Department of Traumatology, BG Trauma Center, University of Tübingen, Schnarrenbergstr. 95, 72076 Tübingen, Germany

**Keywords:** BMSC-exo, deoxynivalenol, oxidative stress, lipid peroxidation, oxylipin, hepatic damage

## Abstract

Deoxynivalenol (DON) is a kind of Fusarium toxin that can cause a variety of toxic effects. DON is mainly metabolized and detoxified by the liver. When the concentration of DON exceeds the metabolic capacity of the liver, it will trigger acute or chronic damage to the liver tissue. Previous studies demonstrated that bone marrow mesenchymal stem-cell-secreted exosomes (BMSC-exos) reduce liver injury. Therefore, we issue a hypothesis that in vitro-cultured rat BMSC-secreted exos could ameliorate liver damage after 2 mg/kg bw/day of DON exposure. In total, 144 lipids were identified in BMEC-exos, including high polyunsaturated fatty acid (PUFA) levels. BMSC-exos treatment alleviated liver pathological changes and decreased levels of alanine aminotransferase, aspartate aminotransferase, inflammatory factors interleukin-6 (IL-6), tumor necrosis factor-α (TNF-α), and lipid peroxidation. Otherwise, low or high BMSC-exos treatment obviously changes DON-induced hepatic oxylipin patterns. According to the results from our correlation network analysis, Pearson correlation coefficient analysis, and hierarchical clustering analysis, the top 10% oxidized lipids can be classified into two categories: one that was positively correlated with copper–zinc superoxide dismutase (Cu/Zn SOD) and another that was positively correlated with liver injury indicators. Altogether, BMSC-exos administration maintained normal liver function and reduced oxidative damage in liver tissue. Moreover, it could also significantly change the oxylipin profiles under DON conditions.

## 1. Introduction

DON is one of the most important trichothecenes in grain contamination, frequently contaminating food and feed around the world [1,2]. The presence of DON in human or animal food can cause serious health problems. For example, 50 μg/kg bw (ip or oral) can elicit emesis in Yorkshire barrows pigs, causing LD50 values for the ip and oral exposure of mice to range from 49 to 70 and 46 to 78 mg/kg bw, respectively. Norwegian Landrace pigs consuming 2 and 4 ppm of DON caused decreased feed intake, weight gain, and feed utilization efficiency throughout the experiment, and short-term exposure to DON at 2.5 and 5 mg/kg bw could impair reproduction and development in rodents [3,4,5]. Therefore, long-term low-dose DON exposure may become a global threat to human health [6].

Several studies have illustrated that DON-induced reactive oxygen species (ROS) production occurs in a dose-dependent manner in in vitro experiments, with a peak of ROS production within 1 h, clearly indicating that oxidative stress is an early event in the process of DON-induced toxic effects [7,8,9]. ROS can initiate lipid peroxidation in lipid membranes, leading to damage to membrane phospholipids and lipoproteins that further trigger membrane dysfunction and indirectly induce DNA and protein denaturation through free radical propagation chain reactions [10,11]. Many studies have also shown that DON exposure could increase lipid peroxide levels and promote the formation of aldehyde products in DON-induced cytotoxicity and apoptosis [7,9,12]. This also suggests that the oxidative damage from DON may be related to its polar chemical structure containing three free hydroxyl groups (•OH) [13]. In addition, according to the 2011 WHO report, humans’ average daily intake of DON is 0.2–14.5 μg/kg bw/day [14]. In some countries, DON intake in children exceeds 1.0 μg/kg bw/day, which is the provisional maximum tolerated daily intake (PMTDI, PDI) [15]. However, the potential harm of this low-dose exposure in target organs remains poorly controlled, especially in the liver, which is mainly responsible for detoxifying DON in animals and humans. Therefore, we hope to find a potential strategy to combat DON-induced liver damage.

Emerging studies have also focused on the therapeutic effects of bone marrow mesenchymal stem-cell-secreted exosomes (BMSC-exos) on liver injury [16,17,18,19]. BMSC-exos are nano-scale vesicles with a lipid bilayer membrane structure of 40–160 nm, which can be separated by the affinity membrane technique, and their membrane lipid composition is closely related to BMSC cells [20]. BMSC-exos have the advantages of low cost, less invasiveness, convenient isolation, easy storage, low immunogenicity among heterogeneous species, and differentiated pluripotency [21,22]. In direct or indirect studies, vesicles from BMSC could conflict with hepatic failure induced by D-galactosamine/tumor necrosis factor-α (TNF-α) [16]. Additionally, human BMSC-exos attenuated rat liver fibrosis by reducing alpha-smooth muscle actin (α-SMA) expressions to restore liver function [23]. Mouse BMSC-exos obviously alleviated autoimmune hepatitis-related liver necrosis and inflammatory markers [24]. Therefore, BMSC-exos may be an ideal strategy for ameliorating liver injury after DON exposure. However, the underlying mechanism of its therapeutic effect is unclear.

Otherwise, many studies of exosomes mainly focused on their contents, such as mRNA, microRNA, and proteins [25,26]. Lipids are an essential part of exosome membranes. Most studies on exosome lipids focused on lipid membrane structure, lipid species, and lipid abundance distribution [27]. Various studies have shown that oxidative stress is often closely associated with the occurrence and progression of liver disease [28,29,30], and antioxidant therapy has been used to treat liver disease [31,32]. Biofilms contain many PUFAs and the unsaturated double bonds in PUFAs are the primary attack targets from free radicals. The particle size of exosomes generally ranges between 40 and 160 nm [20]. The petite particle size makes them more preferentially react with free radicals and induces lipid peroxidation within in vitro experiments [33]. Therefore, according to the above context, we issue a hypothesis that the BMSC-exos lipid membrane might be “another helpful defensive line” against the liver’s oxidative damage with DON treatment.

In this study, we first aimed to evaluate the protective effect of different doses of BMSC-exos on DON-induced liver damage. Next, we explored whether the lipids of BMSC-exos play a critical role in conflicting oxidative damage triggered by DON. We hope that this research can provide a new strategy for future research.

## 2. Materials and Methods

### 2.1. Reagents

DON (12, 13-epoxy-3, 4, 15-trihydroxytrichotec-9-en-8-one, C_15_H_20_O_6_, molecular weight: 296.32, purity ≥ 99%, CAS number: 51481-10-8) was purchased from Sigma-Aldrich (St. Louis, MI, USA). The enhanced BCA protein assay kit (P0010), the total glutathione peroxidase (GPx) kit (S0058), the catalase (CAT) kit (S0051), the total superoxide dismutase (SOD) kit (S0101M), the Cu/Zn-SOD and Mn-SOD kit with WST-8 (S0103), and the lipid peroxidation (MDA) kit (S0131M) were purchased from Beyotime Biotechnology (Shanghai, China). The phosphatase inhibitor cocktail (B15001) and protease inhibitor cocktail (B1400) were purchased from Bimake (Houston, TX, USA). The alanine aminotransferase (ALT) kit (E-BC-K235-M), the aspartate aminotransferase (AST) kit (E-BC-K236-M), and the mouse tumor necrosis factor-alpha (TNF-α) (E-EL-0128c) ELISA kit were purchased from Elabscience Biotechnology Co., Ltd. (Wuhan, China). The mouse interleukin-6 (IL-6) (EK206HS) ELISA kit was purchased from Multisciences (Hangzhou, China). Fetal bovine serum (FBS) and DMEM/F12 media were purchased from Gibco (Thermo Fisher Scientific Inc. Waltham, MA, USA). Exosome-depleted FBS was purchased from SBI (System Biosciences, California, CA, USA). The mesenchymal stem cell surface marker detection kit (RAXMX-09011) was purchased from OriCell Biotechnology Co., Ltd. (Guangzhou, China). DiR iodide [1,1-dioctadecyl-3,3,3,3-tetramethylindotricarbocyanine iodide] cell membrane labeling solution was purchased from AAT Bioquest, Inc. (Sunnyvale, CA, USA). The primary antibodies of CD9 (ab223052), CD63 (ab217345), and CD81 (ab109201) were purchased from Abcam (Cambridge, UK). The secondary antibodies of horseradish peroxidase (HRP)-linked anti-rabbit IgG (7074) and horseradish peroxidase (HRP)-linked anti-mouse IgG (7076) were purchased from CST (Cell Signaling Technology, Inc., Danvers, MA, USA).

### 2.2. Animals’ Housing and Treatment

The Animal Care and Use Institutional Committee of Huazhong University of Science and Technology has approved all animal experiments procedures (IACUC Number: S2851). Eight-week-old SPF C57BL/6J male mice weighing 16–22 g were obtained from Beijing Charles River Experimental Animal Technology Co., Ltd., Beijing, China, and were bought and raised in the SPF Animal Laboratory of Huazhong University of Science and Technology. The temperature of the controlled environment was 22 ± 2 °C, the humidity was 60%, the circadian rhythm was 12 h (day and night from 8:00 a.m. to 8:00 p.m.), and food and drink were free.

**Figure 1:**
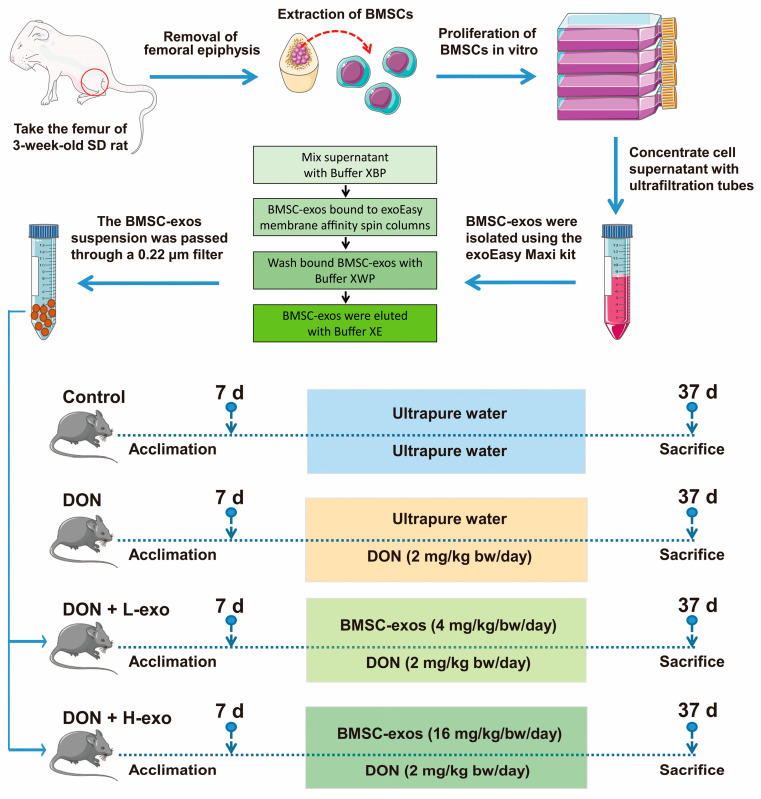
Experimental design and methods. All procedures for stem cell culture, exosome extraction, and experimental grouping and design of this study.

After 1 week of adaptive feeding, the mice were randomly divided into 4 groups: the control group (N = 12), the DON group (N = 12), the DON + L-exo group (N = 12), and the DON + H-exo group (N = 12). The mice in the DON + L-exo group and the DON + H-exo group received 4 mg/kg/bw/day and 16 mg/kg/bw/day of BMSC-exo via an oral gavage, respectively [32]. Then, 2 mg/kg bw/day DON doses were also administered to mice in the DON group, the DON + L-exo group, and the DON + H-exo group via an oral gavage for 30 days [3,34] (Figure 1). The control group mice received the same standard ultrapure water with the gavage, and the mice were sacrificed and dissected immediately after the experiments were conducted. The liver samples were fixed in 4% phosphate-buffered paraformaldehyde for 48 h for hematoxylin–eosin (H&E) staining. The remaining liver samples were rapidly frozen in liquid nitrogen and stored at −80 °C for subsequent experiments.

### 2.3. Exosome Isolation and Characterization

#### 2.3.1. Bone Marrow Stem Cell Culture and Identification

The cell culture conditions and animal surgery strategies were based on previous studies with minor modifications [35,36]. The 3-week-old SPF SD male rats, weighing 60–90 g, were obtained from Beijing Charles River Experimental Animal Technology Co., Ltd. Bone marrow stem cells (BMSCs) were extracted from the femoral bone marrow of the rat. After the cells were washed with the DMEM/F12 medium (supplemented with 10% FBS and 1% (*v*/*v*) penicillin–streptomycin), the suspension of bone marrow was transferred into a 75 cm^2^ cell culture flask and incubated at 37 °C in a humidified atmosphere of 5% CO_2_. The medium was renewed every two days until the cells reached 90% confluency. The BMSC identification experiment was performed according to the instructions of the mesenchymal stem cell surface marker detection kit (RAXMX-09011). Briefly, BMSCs were harvested and suspended in 1 × PBS buffer containing 0.1% BSA. The cell suspension was mixed with trypan blue at a volume of 1:1 and counted by an automatic cell counter (Shanghai Ruiyu IC1000). The cell concentration was then adjusted to 3 × 10^6^ cells/mL. BMSCs were incubated with anti-rat CD29 (1:50; Cyagen Biosciences, California, CA, USA), anti-rat CD44 (1:50; Cyagen Biosciences, Santa Clara, CA, USA), anti-rat CD90 (1:50; Cyagen Biosciences, Santa Clara, CA, USA), and anti-rat CD45 (1:50; Cyagen Biosciences) for 30 min at 4 °C. After the cells were washed twice by the buffer, the fluorescent secondary antibody FITC (1:50; Cyagen Biosciences) was added and incubated at 4 °C for 30 min. After the cells were washed twice with buffer, the percentage of positively stained cells was analyzed by a NovoCyte flow cytometer (ACEA Biosciences, Santiago, California, CA, USA) and NovoExpress™ software (NovoCyte, Santiago, CA, USA).

#### 2.3.2. Exosome Isolation, Purification, and Identification

In this study, BMSC-exos were produced from 3–5 passage BMSCs. After reaching 90% confluency, BMSCs were washed with PBS. After 48 h incubation with the DMEM/F12 medium containing 5% extracellular-vesicle-depleted FBS, the supernatant was collected and centrifuged at 2000× *g* for 20 min to remove the cell debris. According to the manufacturer’s instructions, BMSC-exos were isolated and purified from cell supernatants using the exoEasy Maxi kit (Qiagen, Valencia, CA, USA). Briefly, cell supernatants were filtered using 0.45 μm filters (EMD Millipore, Burlington, MA, USA). An ultrafiltration membrane (MWCO) (Millipore, USA) with a molecular weight cutoff of 100 KDa was concentrated at 1000 × g for 15 min. The supernatant was mixed with Buffer XBP and bound to exoEasy membrane affinity spin columns. Bound BMSC-exos were washed with buffer XWP, eluted with 400 μL of buffer XE (aqueous buffer mainly containing inorganic salts), and the BMSC-exos suspension was filtered through a 0.22 μm filter (Millipore). The protein content was determined with the BCA protein assay kit and stored at −80 °C for further studies, and the final concentration was adjusted to 16 mg/mL. All procedures for stem cell culture and exosome extraction are illustrated in Figure 1.

The morphology of BMSC-exos was observed using a transmission electron microscopy (FEI Tecnai 12, Philips, Holland). Then, 10 μL of uranyl acetate was added dropwise with a pipette onto the copper mesh for 1 min and the suspension was removed with filter paper. After drying for several minutes at room temperature, electron microscopy was performed at 100 kV to obtain the TEM imaging results.

Meanwhile, after diluting the exosome suspension 100-fold, the scattered light signal of BMSC-exos particles was collected through nanoparticle tracking analysis (NTA; NanoSight NS300, Malvern, UK). Three 60 s images were then repeatedly shot, sampling 30 frames per second. The NTA 3.3 Dev Build 3.3.104 software tracks and each particle’s Brownian motion were used to calculate the nanoparticle’s hydrodynamic radius and concentration.

### 2.4. Western Blotting

The protein markers (CD9, CD63, and CD81) of BMSC-exos were detected by Western blot. Protein samples of 20 μg were loaded and separated through 12% SDS-PAGE gel. Proteins were then transferred to 0.45 μm nitrocellulose (NC) membrane (Millipore, HATF00010, USA), followed by blocking for 1 h using 5% nonfat milk in TBST. The membrane was incubated into the primary antibody with a 1:1000 dilution overnight at 4 °C. For detection purposes, the membranes were incubated with the secondary antibody of peroxidase-conjugated horseradish for 1 h. The NC membrane was sprayed with ECL staining solution into a gel imaging analyzer (Syngene, Cambridge, UK), the images were collected by GeneSnap (Syngene, Cambridge, UK), and images were analyzed by GeneTools (Syngene, GeneTools 4.0, Cambridge, UK).

### 2.5. Liver Function Detection

The serum ALT and AST levels in four groups of mice (N = 9–12) were quantified in triplicate using commercial kits (Elabscience, Wuhan, China). Test results are expressed as IU/L. A Syngene multimode reader (BioTek Instruments, Winooski, VT, USA) was used to measure the optical density (OD) values.

### 2.6. Systemic Inflammatory Cytokine Detection

According to the manufacturer’s instructions, four groups of mice serum IL-6 and TNF-α levels (n = 9–12) were duplicated using commercially available ELISA kits. The test results were expressed in pg/mL. A Syngene multimode reader measured the optical density (OD) values (BioTek Instruments, USA).

### 2.7. Determination of Lipid Peroxidation Levels and Antioxidant Enzyme Activities in the Liver

Then, 10% mouse liver homogenate was prepared with a high-throughput tissue grinder (Xinzhi Biotechnology Co., Ltd. Ningbo, China). The homogenate of liver tissue was centrifuged for 10 min at 2500 rpm/min to remove the precipitate. The D liver MDA levels and antioxidant enzyme activities (including total SOD, Cu/ZnSOD, MnSOD, CAT, and GPX) across four groups were measured in triplicate according to the manufacturer’s protocol (Beyotime, Hangzhou, China). The liver 4-HNE content of four groups of mice was determined in duplicate using a commercially available ELISA kit (Elabscience, Wuhan, China). Through a multi-plate reader (Bio-Tek, USA), optical density (OD) values were measured. Indicator levels were finally normalized to every milligram of protein.

### 2.8. Exosome Labeling and Tracking Distribution in Mice

Then, a 5 µM final concentration of DiR was co-incubated with BMSC-exos at 37 °C for 30 min in the dark. Then, DiR-labeled BMSC-exos were washed with PBS and passed through the exosome extraction procedure again to remove the excess dye. Mice were scanned at 745 nm (λ_ex_) using an exposure time of 300 ms per image frame after the gavage of DiR-labeled BMSC-exos. The DiR-labeled BMSC-exos were tracked in mice using the SPECTRAL Lago X in a vivo imaging system [37].

### 2.9. Analysis Profiles of Lipids and Hydroxyl Lipids

#### 2.9.1. Lipid Profiling

Untargeted lipidomics analysis was performed based on previous research [38]. The lipid profiling of BMSC-exos samples was performed using the LC-MS/MS method with a Vanquish UHPLC system (Thermo Fisher, Germany) and an Orbitrap Q ExactiveTM HF mass spectrometer (Thermo Fisher, Germany) was obtained. The methanol (0.75 mL) was added to the exosome sample (100 µL). MTBE (2.5 mL) was then added and the mixture was incubated on a shaker for 1 h at room temperature. The addition of water (0.625 mL, MS-grade) induced the phase separation. After incubation at room temperature for 10 min, the sample was centrifuged at 1000× *g* for 10 min. The upper (organic) phase was harvested, and the lower phase was back-extracted with 1 mL of solvent mixture (MTBE/methanol/water, 10:3:2.5, *v*/*v*/*v*) and the upper phase was managed. After drying, the composite organic phase was dissolved in isopropyl alcohol (100 μL) and stored as well as analyzed by LC-MS/MS [39]. Compound Discoverer 3.01 (CD3.1, Thermo Fisher) was used to process the original data files generated by UHPLC-MS/MS. The peak comparisons and peak selections of each metabolite were performed, and they were then quantified. The main parameter settings were as follows: a retention tolerance of 0.2 min, an actual quality tolerance of 5 ppm, a signal strength tolerance of 30%, a signal-to-noise ratio of 3, and a minimum strength of 100,000. After that, the peak intensity was normalized to the total spectral intensity. Using normalized data, molecular formulas were predicted based on additive ions, molecular ion peaks, and fragment ions. Then, the LipidMaps [40] and LipidBlast [41] were used for peak matching to obtain accurate qualitative and relative quantitative results.

The chromatographic conditions were as follows: Thermo Accucore C30 (150 × 2.1 mm, 2.6 μm) column, an injection gradient of 20 min, and a flow rate of 0.35 mL/min. The column temperature was set at 40 °C. Mobile phase buffer A consisted of acetonitrile–water (6/4) + 10 mm of ammonium acetate + 0.1% formic acid and mobile phase buffer B consisted of acetonitrile–isopropyl alcohol (1/9) + 10 mm of ammonium acetate + 0.1% formic acid. The solvent gradients were initially set as 30% B 2 min, 43% B 5 min, 55% B 5.1 min, 70% B 11 min, 99% B 16 min, and 30% B 18.1 min.

Q ExactiveTM HF mass spectrometry operated in the positive (negative) polarity mode with the following conditions: jacket gas—20 psi, scavenging gas—1 L/min, auxiliary gas rate—5 L/min (7 L/min), spray voltage—3 kV, capillary temperature—350 °C, heater temperature—400 °C, S-Lens RF level—50, scanning range—140–1700 *m*/*z*, automatic gain control target—1 × 10^6^, positive collision energy—25 eV and 30 eV (20 eV, 24 eV, and 28 eV), injection time—100 ms, isolation window—1 *m*/*z*, automatic gain control objective (MS2)—1 × 10^5^, and dynamic exclusion—15 s.

#### 2.9.2. Oxylipin Profiling

Oxylipidomics analysis was performed based on previous research [42]. The lipids from liver samples were analyzed by LC-MS/MS using the ExionLC™AD system (SCIEX) and the QTRAP^®^6500+ mass spectrometer (SCIEX). Then, 2 mg doses of liquid nitrogen ground tissue samples were taken, diluted to 1 mL with 50 mM phosphate buffer, and then equilibrated using a Strata-X reversed-phase SPE column, eluted with methanol (3 mL) and equilibrated with 3 mL of MS water. After loading, the samples were eluted with 10% methanol (3 mL) to remove any impurities. Eluted metabolites were added with methanol (1 mL), dried with a nitrogen blower, and a resolvent (water–acetonitrile–acetic acid in a volume ratio of 60:40:0.02) was added to dissolve for 5 min and then placed in a centrifuge tube of 15,000× *g*. After centrifugation at 4 °C for 10 min, the supernatant was collected and injected into LC-MS for analysis [43]. An equal amount of each sample was taken from each experimental sample and mixed as the QC sample. SCIEX OS V1.4 software was used for the peak integration and calibration of original data. The peak area of each chromatographic peak represents the relative quantitative value of the relevant material. The integrated peak area averages were compared to each of the lipid conditions.

Chromatographic conditions: C18 column (10 cm × 2.1 mm) was injected with a linear gradient of 14 min at a flow rate of 0.3 mL/min. The eluents were eluent A (0.1% formic acid) and eluent B (acetonitrile). The solvent gradients were set as 35% B 0.5 min, 35–95% B 9.5 min, 95% B 10.5 min, 95–35% B 11 min, and 35% B 14 min [44].

The QTRAP^®^6500+ mass spectrometer operated in a positive polarity mode with a screen gas of 40 psi, a collision gas as the medium, an ion spray voltage of −4500 V, a temperature of 500 °C, an ion source gas of 1:55, and an ion source gas of 2:55.

### 2.10. Statistical Analysis

The preprocessed oxylipidomics dataset was imported into MetaboAnalyst for orthogonal projections to latent structure–discriminate analysis (OPLS-DA), volcano plot analysis, and principal component analysis (PCA). The variable importance in projection (VIP) score was > 1 (*p* < 0.05) and the fold change (FC) was >2 (upregulated) or <0.5 (downregulated) to screen for metabolite species that significantly differed between groups. Pathway enrichment analysis and metabolite annotation were performed using the Kyoto Encyclopedia of Genes and Genomes (KEGG) metabolic pathways database. Prism software (GraphPad 8.0) was used for data processing and statistical analysis. All experimental results are expressed as mean ± standard error (x ± SE). All data were derived from more than three separate experiments. One-way analysis of variance (ANOVA) was used to compare the multiple groups. Significance was defined as *p* < 0.05. For detailed lipid detection methods, please refer to the “Materials and Methods S1”.

## 3. Results

### 3.1. Characterization of BMSCs and BMSC-exos

**Figure 2:**
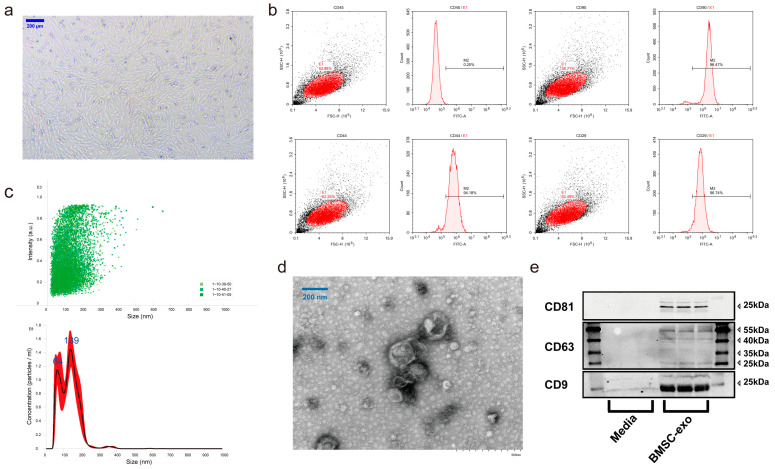
Isolation and characterization of BMSCs and BMSC-exos. (**a**) BMSCs again exhibited a typical spindle-shaped and fibroblast-like morphology in the view. The scale bar is 100 μm. (**b**) BMSC-specific surface markers such as CD45, CD90, CD44, and CD29 were measured by flow cytometry. (**c**) Determination of BMSC-exos particle size using NTA. (**d**) TEM observation of BMSC-exos morphology. (**e**) Western blot of exosome biomarkers.

The morphology and surface marker expression characteristics of BMSCs in this study conformed to the mesenchymal stem cell criteria defined by the International Society for Cell Therapy (ISCT), which could satisfy subsequent experiments [45]. After three passages, the BMSC cells were fibroblast-like, and most cells had clear borders and formed uniform colonies (Figure 2a). BMSC-specific biomarkers were identified by flow cytometry, and the data showed expressions of positive markers of CD29, CD44, and CD90, but a negative expression of CD45 could also be observed (Figure 2b).

Moreover, with the use of TEM, the BMSC-exos were observed to have a typical cup-like morphology (Figure 2c). NTA showed that the size of BMSC-exos was predominantly distributed between 30 and 200 nm in size (Figure 2d). The bands of exosome-specific markers CD63, CD9, and CD81 in BMSC-exos were also observed. In contrast, the above proteins were not detected in the cell culture media, indicating the successful isolation of high-purity BMSC-exos (Figure 2e).

### 3.2. Comprehensive Analysis of Lipid Profiles for BMSC-exos Using UHPLC-MS/MS

**Figure 3:**
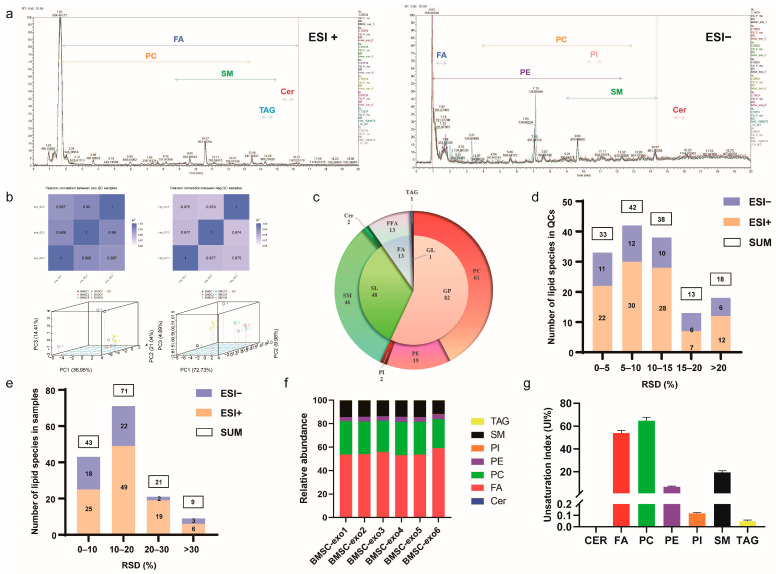
Lipidomics analysis of BMSC-exos samples. (**a**) Total ion chromatograms (TICs) of lipid profiles in BMSC-exos were obtained using UHPLC-MS/MS (ESI+ and ESI−). (**b**) Correlation between QC samples and PCA analysis of all samples, including QCs. (**c**) Lipid classes and species distributions identified from BMSC-exos samples. (**d**) RSD distribution of lipid species detected in all QC samples (n = 3). (**e**) RSD distribution of lipid species detected in different BMSC-exos samples (n = 6). (**f**) Distribution of lipid species and abundance in BMSC-exos. (**g**) Unsaturation index (UI) of different lipid classes in BMSC-exos.

Untargeted lipidomic analysis for BMSC-exos using UHPLC-MS/MS enables the unbiased analysis of almost all classes of lipids in samples. The UHPLC system in ESI+ and ESI− modes was able to separate and elute various polar lipids in BMSC-exos within 20 min (Figure 3a). We identified lipid species with stringent criteria. Firstly, the parameters such as retention time and mass-to-charge ratio were screened. For different samples, peak alignment was performed according to the retention time and mass deviation to make the identification more accurate. Peak extraction and peak area quantification were performed according to the set ppm, signal-to-noise ratio, adduct ions, and other information. The qualitative and relative quantitative results of lipids were obtained by searching and comparing Lipidmaps and Lipiblast spectral databases. Finally, a total of 144 lipids were identified in BMSC-exos samples, including 13 free fatty acids (FFAs), 1 triacylglycerol (TAG), 61 phosphatidylcholines (PCs), 19 phosphatidylethanolamines (PEs), 2 phosphatidylinositols (PIs), 46 sphingomyelins (SMs), and 2 ceramides (Cers) (Figure 3c).

To ensure the reproducibility and stability of the BMSC-exos lipidomics method, we calculated the Pearson correlation coefficient among quality control (QC) samples. The higher the correlation of QC samples (R2 is closer to 1), the better the stability and data quality of the whole detection process (Figure 3b). The total ion chromatogram (TIC) plots of six BMSC-exos samples and multiple QCs were overlaid (Figure 3a). Meanwhile, unsupervised principal component analysis (PCA) was performed on all samples, including QCs, which showed that all QC samples were tightly clustered (Figure 3b). We also evaluated the relative standard deviation (RSD) distribution of 144 lipids in all QC samples (Figure 3d). Moreover, data demonstrated that approximately 79% of the lipids (113) had an RSD of less than 15%. The above pieces of evidence showed that the UHPLC-MS/MS method was reproducible throughout the experimental period. Furthermore, we evaluated the RSD distribution of 144 lipids in BMSC-exos samples by extracting from different batches (Figure 3e), and approximately 79% of lipids (114) had less than 20% RSDs. At the same time, the abundances of different lipids were uniformly distributed in different BMSC-exos samples (Figure 3f). The above results showed that the BMSC-exos samples used in this experiment had good stability and consistency.

To assess the degree of lipid unsaturation in BMSC-exos, we also quantified the unsaturation index (UI). The degree of unsaturation was calculated as an index of the sum of each lipid’s relative contents, multiplied by their respective number of double bonds [46,47]. BMSC-exos contained a large amount of polyunsaturated fatty acids (PUFAs), of which phosphatidylcholine (PC) had the highest degree of unsaturation, with a UI of 65% (Figure 3g).

### 3.3. BMSC-exos Alleviated DON-Induced Liver Damage

**Figure 4:**
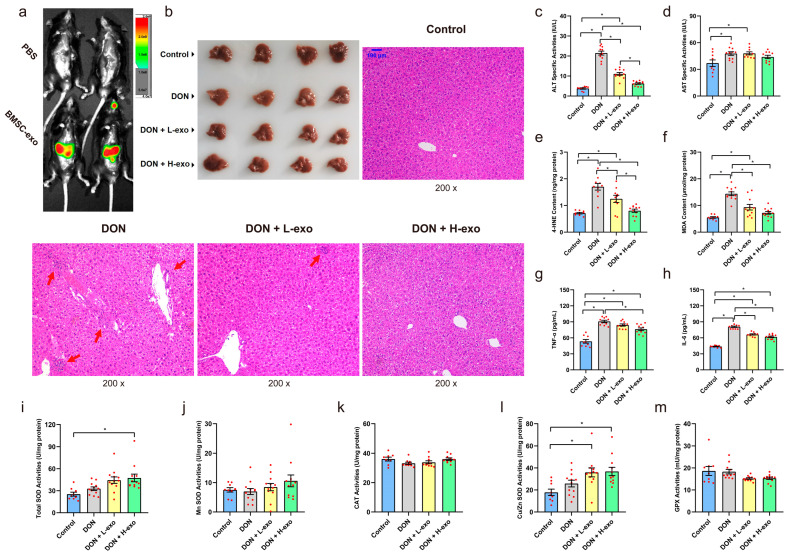
BMSC-exos attenuate DON-induced liver injury. (**a**) Three hours after oral gavage, in vivo fluorescence imaging was used to observe the distribution of DiR-labeled BMSC-exos in DON-infected mice. (**b**) Representative optical and H&E staining images of the livers of four groups of mice after experimental treatment, the red arrow points to lymphocyte infiltration, and the scale bar is 100 μm. (**c**–**h**) The liver function indexes, oxidative stress product content, and systemic inflammatory cytokines in the four groups of mice after experimental treatment. (**i**–**m**) Antioxidative enzyme activity in the liver of four groups of mice. Data are expressed as mean ± SE; “*” means *p* < 0.05. The DON + L-exo group received 4 mg/kg/bw/day of BMSC-exos + 2 mg/kg bw/day of DON and the DON + H-exo group received 16 mg/kg/bw/day of BMSC-exos + 2 mg/kg bw/day of DON.

After the oral gavage of DiR-labeled BMSC-exos for 3 h, DiR signals could be detected in the upper abdomen of mice. Meantime, no background fluorescence was detected in PBS control mice, confirming that the signal in treated animals was from DiR-labeled BMSC-exos (Figure 4a). Otherwise, we sacrificed the animals, excised major organs for ex vivo imaging, and observed DiR signals in the liver to confirm that BMSC-exos reached the liver (Figure S1). Histological examination showed the prominent infiltration of inflammatory cells around the central vein of liver lobules in DON group mice, accompanied by deformation, the swelling of liver cells, and the irregular arrangement of hepatic cords. However, BMSC-exos administration alleviated the hepatocyte injury mentioned above (Figure 4b). Compared with the control group, the serum ALT, AST, IL-6, and TNF-α levels of mice in the DON group were significantly increased. The BMSC-exos treatment at 16 mg/kg bw/day could significantly inhibit the serum ALT, IL-6, and TNF-α levels. Meanwhile, the levels of MDA and 4-HNE in the liver of mice were significantly increased after DON exposure, but BMSC-exos could significantly decrease the levels of lipid peroxidation in the liver (Figure 4c–h). In addition, SOD and Cu/Zn SOD activities were increased after BMSC-exos administration (16 mg/kg bw/day). However, CAT and GPX activities were not significantly changed (Figure 4i–m).

### 3.4. Effects of BMSC-exos Administration on Oxylipin Profile or Pattern after DON Exposure

**Figure 5:**
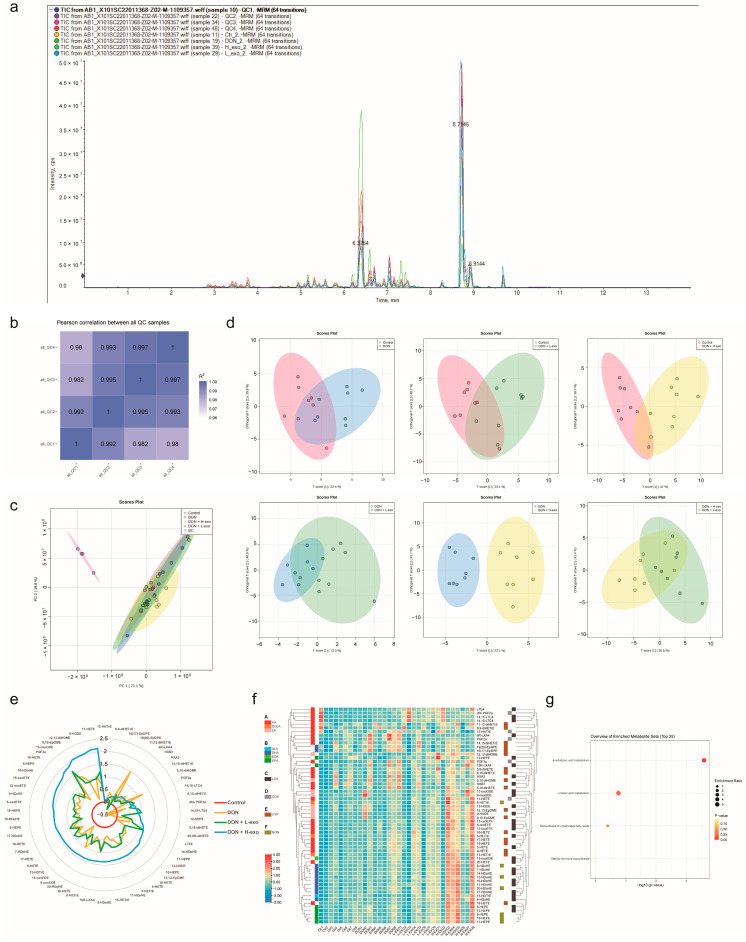
Oxidized lipidome of the liver. (**a**) Total ion chromatogram (TIC) of oxidized lipid profile in the liver. (**b**) Correlation between QC samples. (**c**) PCA analysis of all samples, including QCs. (**d**) Orthogonal partial least squares discriminant analysis (orthoPLS-DA) of the relative oxylipins content in mouse liver between different groups. (**e**) Radar plot showing oxylipin distribution patterns, with data expressed as log(2) FC over control. (**f**) Hierarchical clustering analysis of the relative abundance distribution heat map of oxylipins in four groups of mice according to the precursor source of oxidized lipid and the oxidation reaction pathway. (**g**) Pathway enrichment analysis of the oxidized lipidome. All matched pathways are plotted according to the *p*-value from pathway enrichment analysis and pathway impact values from pathway topology analysis. The color gradient and circle size indicate the significance of the pathway ranked by *p*-value (yellow, higher *p* values; red, lower *p* values) and pathway impact values (the larger the circle, the higher the impact score).

The results of oxidized lipid profiles demonstrated 62 oxylipins in the liver with HPLC-MS/MS analysis. This method can simultaneously explore many oxygenated PUFA species with high sensitivity and specificity. The HPLC system could separate and elute various oxylipins in the liver within 10 min (Figure 5a). We qualitatively analyzed the compounds in terms of Q1 (precursor ion), Q3 (product ion), RT (retention time), DP (declustering potential), and CE (collision energy). Compounds were quantified based on the peak area of Q3 (product ion). Mass detection analysis TIC plots of different samples and multiple QCs were overlaid (Figure 5a), the Pearson correlation coefficient R2 was close to 1 between QC samples (Figure 5b), and the QC samples in PCA analysis (shown as pink dots) was centrally aggregated (Figure 5c), which meant that the extraction and detection process of oxylipins in this study had excellent system reproducibility and stability. The orthogonal partial least squares discriminant analysis (OPLS-DA) model is a supervised discriminant analysis method used to investigate the differentially oxidized lipidome further. The abscissa directions of the DON group and the DON+H-exo group were utterly separated from each other in the OPLS-DA score graph, which meant that there were significantly different oxylipin profiles between the two groups. However, the OPLS-DA score maps of the other pairwise combinations were not completely separated in the abscissa direction (Figure 5d). This indicates that the administration of 16 mg/kg bw/day of BMSC-exos could significantly change the DON-induced hepatic oxylipin profile. A radar chart displayed the distribution pattern of oxylipins in the liver, and the data were expressed as log(2) FC over control (Figure 5e). Compared with the control group, most of the oxylipins in the DON group increased, and only a tiny part decreased. Compared with the DON group, most oxylipins were further elevated after BMSC-exos administration.

**Figure 6:**
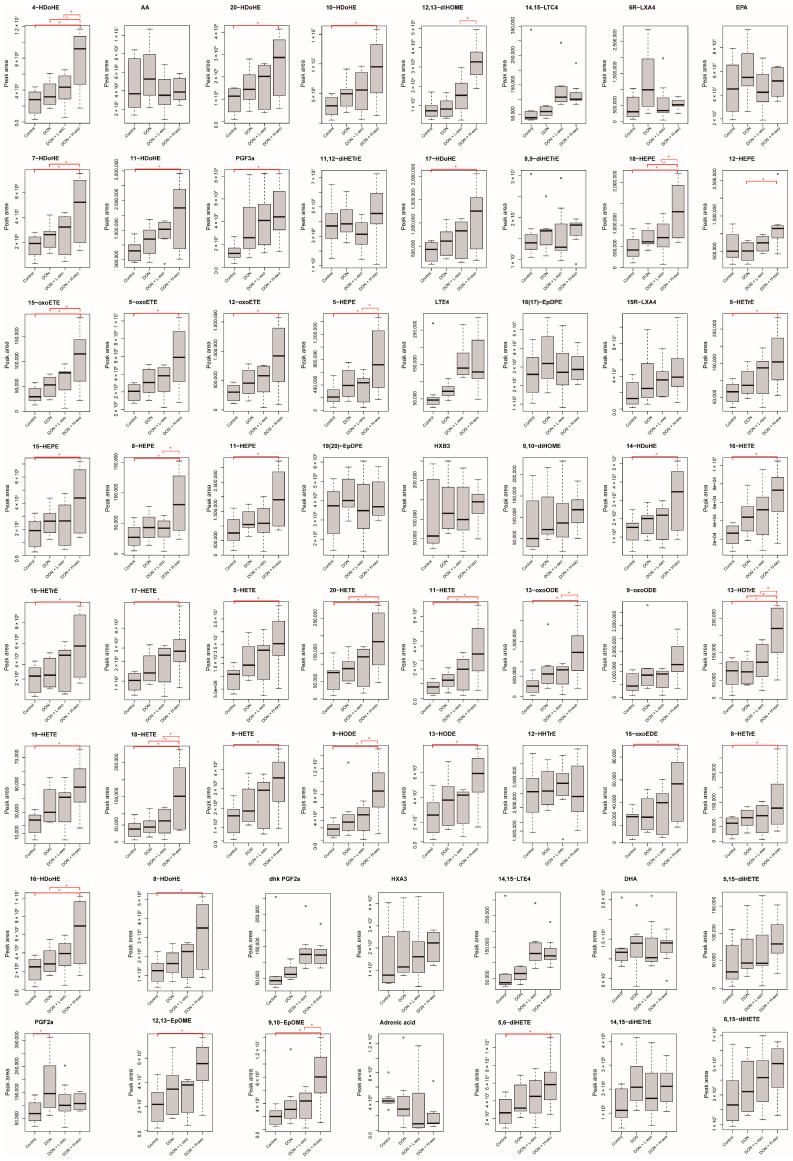
Boxplot of the relative content of all oxylipins detected in this study among the four groups. “*” means *p* < 0.05.

PUFAs are direct metabolic precursors for lipoxin biosynthesis, and they belong to two prominent families: omega-3 PUFAs (ALA, DHA, EDA, and EPA) and omega-6 PUFAs (AA, DGLA, and LA). Enzymatic and/or non-enzymatic reactions oxidize polyunsaturated fatty acids, and the three main enzymatic pathways involved in lipoxin production are cyclooxygenase (COX), lipoxygenase (LOX), and cytochrome P450 (CYP) isomer catalysis. Hierarchical clustering analysis of the heat map of relative abundance distribution of oxylipins showed that the distribution characteristics of oxylipin abundance in different groups were more related to the source of precursor lipids but less related to the formation pathway through enzymatic or non-enzymatic processes (Figure 5f). The enrichment analysis showed that the oxylipins detected in the liver were mainly enriched in the arachidonic acid metabolic pathway and the linoleic acid metabolic pathway (Figure 5e). KEGG pathway annotation was performed on the detected oxylipins using Pathview (Figure S2) to better observe changes in the detected oxylipins in the above two pathways. In order to more intuitively view the changing trend of each oxylipin in the four groups, we made a boxplot analysis of the relative content of all detected oxylipins in the four groups (Figure 6).

### 3.5. Screening for Different Key Oxylipins

**Figure 7:**
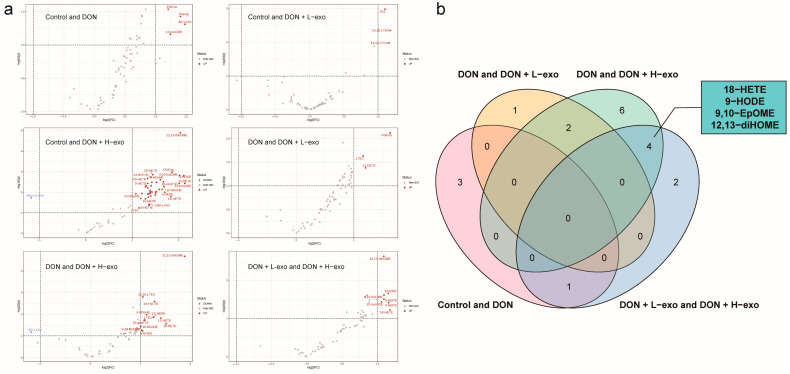
Screening of differential oxylipins with critical roles. (**a**) Volcano plot analysis to screen for significantly different metabolites between different groups. Red circles indicate significantly up-regulated differential metabolites and blue circles indicate significantly down-regulated differential metabolites. The fold change is log-transformed, and the farther it is from the coordinate origin (0, 0), the more significant the feature is. (**b**) Venn diagram for further the screening of differential oxylipins with critical roles.

Volcano plot analysis was used to screen for the metabolites that differed significantly between groups, with *p* < 0.05 and a fold change (FC) > 2 (up-regulation) or < 0.5 (down-regulation) selected as important features (Figure 7). The differential metabolites of “VIP > 1” between different groups (Table S1) were analyzed using a Venn diagram to obtain the intersection. The differential metabolites of “Control and DON” and “DON and DON + L-exo and DON and DON + H-exo” did not intersect, indicating that BMSC-exos could defend against free radicals through their PUFAs, rather than altering the contents of mouse liver to alter the DON pattern oxylipin profile (Figure 7). The 18-HETE, 9-HODE, 9,10-EpOME, and 12,13-diHOME in the intersection represent the DON-mode oxylipins that BMSC-exos could alter substantially in a dose-dependent manner (Figure 7).

### 3.6. Association of Key Oxylipins with Lipid Peroxidation, Antioxidants, and Liver Function/Phenotype

**Figure 8:**
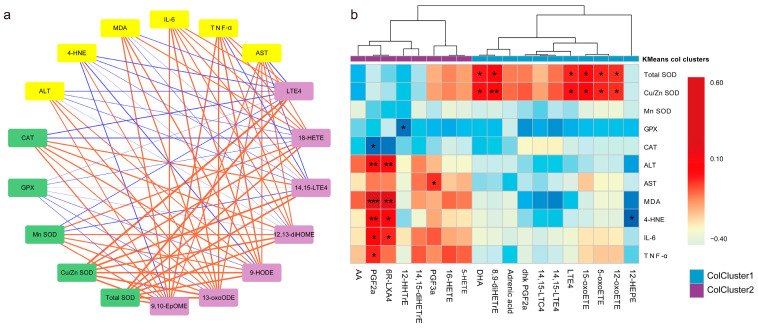
Association analysis of oxylipins and liver phenotypes. (**a**) Correlation network between differential oxylipins and liver biochemical indicators. The red line indicates a positive correlation and the blue line indicates a negative correlation. The thicker lines represent stronger correlations. (**b**) Screen oxylipins with strong phenotype correlation perform hierarchical clustering. “*” means *p* < 0.05, “**” means *p* < 0.01, “***” means *p* < 0.001.

The correlation network between the seven oxylipins (screened in Section 3.5) and liver biochemical indexes was constructed by calculating the Pearson correlation coefficient (Figure 8a). Among them, Cu/Zn SOD had the strongest and positive correlation with the targeted oxylipins, indicating that Cu/Zn SOD played a synergistic role in the process of BMSC-exos changing the DON-mode oxylipin profile. The 4-HNE was negatively correlated with oxylipins, and was specifically altered by BMSC-exos, suggesting that BMSC-exos defense against free radicals may reduce 4-HNE production (Figure 8a). To comprehensively observe the correlation between liver oxylipin profiles and biochemical indicators, we screened the indicators with the top 10% correlation coefficients to construct a heat map. Among them, red represents a positive correlation, blue represents a negative correlation, and asterisks represent a significant correlation. Hierarchical clustering found that these oxylipins were divided into two categories: one that was mainly positively correlated with Cu/Zn SOD and others that were mostly positively correlated with liver function indexes (ALT, AST), inflammatory factors (IL-6, TNF-α), and oxidative stress products (MDA, 4-HNE) (Figure 8b).

## 4. Discussion

Accumulating evidence suggests that oxidative damage is one of the primary mechanisms by which DON induces cytotoxicity or pathological tissue damage [9,48]. Meanwhile, many studies have also shown that the production of ROS in hepatocytes is related to the dose and duration of DON administration. For example, DON (0.1 μg/mL) can induce oxidative stress in rat liver clone-9 cells and its ROS level is further correlated with hepatotoxicity [12]. However, when HepG2 cells were incubated with DON (15–60 μM) for 1 h, intracellular ROS levels were not significantly increased at lower concentrations but increased significantly (1.4-fold) at 60 μM [9,49]. In addition, it has been reported that DON can dose-dependently induce ROS generation, with ROS generation peaking within 1 h [7,8,9]. This indicates that oxidative stress is an early event in the process of DON-induced toxicity.

Other studies have shown that MSC-exos can reverse liver oxidative damage [32]. However, the roles and underlying mechanisms of BMSC-exos in the progression of hepatic oxidative damage are not fully understood. Therefore, we considered oxidative stress as a key point to explore the protective effects of BMEC-exos on hepatotoxicity induced by DON exposure. Otherwise, exosomes can maintain their structure with high tolerance to gastric acid and then enter the liver after oral administration [32,50]. Therefore, this study adopted this non-invasive method for exosome administration purposes. This study basically validates our hypothesis that BMSC-exos can attenuate pathological liver changes by decreasing the levels of lipid peroxidation (MDA and 4-HNE) and inflammatory factors (IL-6 and TNF-α) to further maintain liver function indicators (ALT and AST).

**Figure 9:**
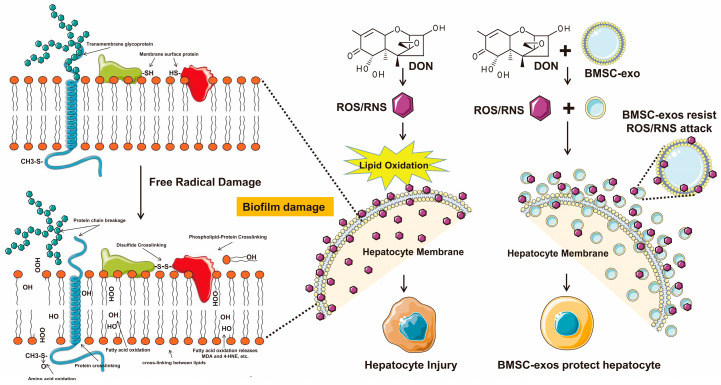
Mechanism mode diagram. The “main battlefield” or “first battle line” of DON-induced lipid peroxidation occurs on exosome membranes, thereby further alleviating the damage of lipid peroxidation to hepatocyte membranes and other cellular components.

PUFAs in organisms mainly exist in the lipids of biological membranes. The non-polar matrix region in the middle of phospholipid bilayers is ideal for free radicals to initiate and propagate amplification chain reactions [51,52,53]. Therefore, biofilms are the primary target of free radicals’ attack. Studies have shown that liposomes with smaller sizes are more vulnerable to free radicals’ attack because of the greater curvature of their bilayer leaflets and wider lipid–lipid spacing [33]. The lipid composition of the membrane also affects the lipid oxidation of liposomes [54]. Exosomes are nano-scale vesicles with a lipid bilayer membrane structure of 40–160 nm, and their membrane lipid composition is closely related to the type of cell from which they are derived [20]. Therefore, we comprehensively analyzed lipid species in BMSC-exos using UHPLC-MS/MS. A total of 144 lipids were identified, containing a large amount of PUFAs, of which PC had the highest degree of unsaturation, with a UI of 65%. This suggests that BMSC-exos have the potential to defend against free radicals preferentially. Since the oxidation of PUFAs by free radicals can generate a series of lipid metabolites, these generated mediators of oxygenated PUFAs play essential roles in the physiological and pathological regulation of many critical biological processes. However, because such mediators may have opposite and redundant properties, it is difficult to fully explain the molecular mechanism of biological processes by only studying a limited number of eicosanoids, and the overall balance between various oxygenated PUFAs regulates many biological processes [55,56]. Therefore, we performed a comprehensive analysis on the oxylipin profile in the liver using HPLC-MS/MS, which enables many oxygenated PUFA metabolites to be simultaneously measured in order to further explore their roles in health and disease conditions. We detected a total of 62 oxylipins in mouse liver. The increasing dose of BMSC-exos could significantly change the DON-induced hepatic oxylipin profile pattern. Compared with the DON group, most oxylipins were further elevated after BMSC-exos administration. Moreover, the distribution characteristics of oxylipin abundance in different groups were closely related to the source of precursor lipids, mainly derived from the metabolism of arachidonic acid and linoleic acid. We screened the oxylipins, with significant differences noted between groups. After taking the intersection of Venn diagram analysis, we found that BMSC-exos resisted free radicals through their PUFAs, rather than changing the contents of the liver to alter the oxylipin profile pattern of DON. These results confirmed our hypothesis: (1) based on the small particle size and higher membrane lipid unsaturation of exosomes, BMSC-exos are preferentially attacked by free radicals than hepatocytes; (2) the “main battlefield” or “first helpful battle line” of DON-induced lipid peroxidation may occur on exosome membranes, thereby further alleviating lipid peroxidation damage to liver cell membranes and other cellular components (Figure 9).

Lipid peroxidation is a continuous chain reaction that produces lipid hydroperoxide (LOOH). Peroxidation can only be stopped when there is a severely damaging effect, a reaction with antioxidant enzymes, or a reaction with antioxidants with a free radical chain blocking effect [57]. Generally, there are three main aspects of cell damage caused by lipid peroxidation: (1) lipid membrane changes lead to membrane dysfunction and membrane enzyme damage; (2) new free radicals generated during the chain reaction can further damage enzymes and other cellular components damage; (3) the effects of LOOH’s decomposition products on cells and their components (especially aldehyde products, such as MDA and 4-HNE) are toxic [58,59,60]. Since the lifespan of ROS is extremely short, as the lifespan of hydroxyl radicals is only 10^−9^ s, free radicals are mainly damaged through lipid peroxidation to cell membrane damage [60,61]. On the other hand, LOOH has a longer lifespan and can exist for several hours to several days before homolysis. The generated aldehyde products can cause damage to spread to other cellular components or even escape to other cells to cause damage [60,62]. It is not enough to replace tissue cells to resist the attack of free radicals and it is necessary to block the chain reaction of lipid peroxidation in time. Therefore, we measured the activity of antioxidant enzymes and found that the activity of Cu/Zn SOD in the liver of mice after BMSC-exos treatment was significantly higher than that of the control group. In addition, Cu/Zn SOD had a strong positive correlation with the screened BMSC-exos function-related targeted oxylipins. At the same time, we screened the top 10% oxylipins with biochemical index correlations for hierarchical clustering and found that these oxylipins were divided into two categories: one was mainly positively correlated with Cu/Zn SOD and the other one was mainly positively correlated with liver injury indexes. This result indicates that Cu/Zn SOD played a synergistic role with BMSC-exos in resisting DON-induced lipid peroxidation and might play a role in terminating the chain reaction. Based on this study, we believe that the preferential reaction of BMSC-exos with free radicals through its lipid membrane is an essential mechanism for inhibiting DON’s toxic effects. The type and antioxidant content of exosomes further determine their effectiveness in resisting oxidative stress damage. Therefore, in future studies, BMSC-exos can also be engineered by encapsulating antioxidant content to further optimize the effect of ameliorating DON-induced oxidative damage.

## 5. Conclusions

In conclusion, this study demonstrated that BMSC-exos exerted a hepatoprotective effect in a dose-dependent manner. The exosomal lipidome and liver oxidized lipidome showed that BMSC-exos defended themselves against free radicals through their PUFAs and could significantly alter the DON-patterned oxylipin profile.

## Data Availability

The data that support the findings of this study are available from the corresponding author upon reasonable request.

## Supplementary Materials

The following supporting information can be downloaded at: https://www.mdpi.com/article/10.3390/antiox12030588/s1. Figure S1: Imaging of ex vivo organs. Figure S2: KEGG pathway annotation of detected oxylipins using Pathview. Table S1: The VIP value of differential oxylipins between groups. Materials and Methods S1: Analysis profiles of lipids and hydroxyl lipids in BMSC-exos.
