# Supplementary material for: Bone Marrow Mesenchymal Stem-Cell-Derived Exosomes Ameliorate Deoxynivalenol-Induced Mice Liver Damage
Source: Antioxidants (Basel). 2023 Feb 27;12(3):588. doi: 10.3390/antiox12030588 (PMC10045494; doi:10.3390/antiox12030588)
Supplement: Supplementary file 1 [file antioxidants-12-00588-s001.zip › Materials and Methods S1.pdf]

Materials and Methods S1. Analysis profiles of lipids and hydroxyl lipids in BMSC-exos

1. Lipid extraction

The methanol (0.75 mL) was added to the exosome sample (100 μL), which was placed into a glass tube with a Teflon-lined cap and vortexed. Then, MTBE (2.5 mL) was added and the mixture was incubated for 1 hour at room temperature in a shaker. Phase separation was induced by adding water (0.625 mL, MS-grade). Upon 10 min of incubation at room temperature, the sample was centrifuged at 1,000 g for 10 min. The upper (organic) phase was collected, and the lower degree was re-extracted with 1 mL of the solvent mixture (MTBE/methanol/water, 10:3:2.5, v/v/v) and managed the upper phase. Combined organic phases were dried and dissolved in isopropanol (100 μL) for storage, then analyzed by LC-MS/MS.

2. Hydroxy lipid extraction

Take 2 mg of liquid nitrogen ground tissue samples, diluted to 1 mL with 50 mM phosphate buffer, then equilibrated using a Strata-X reversed-phase SPE column, eluted with methanol (3 mL) and equilibrated with 3 mL of MS water. After sample loading, impurities were removed by elution with 10% methanol (3 mL). Subsequently, methanol (1 mL) was added to elute the metabolites, the metabolites were blown dry by a nitrogen blower, and a resolvent (water/acetonitrile/acetic acid, volume ratio: 60:40:0.02) was added to dissolve for 5 min, then placed in a centrifuge tube of 15000 g. After centrifugation at 4°C for 10 min, the supernatant was collected and injected into LC-MS for analysis. An equal sample volume was taken from each experimental sample and mixed as a QC sample.

3. Chromatographic and mass spectrometry conditions for lipid and hydroxylipid analysis

Untargeted lipidomics: UHPLC-MS/MS analyses were performed using a Vanquish UHPLC system (Thermo Fisher, Germany) coupled with an Orbitrap Q ExactiveTM HF mass spectrometer (Thermo Fisher, Germany). Samples were injected onto a Thermo Accucore C30 column (150 × 2.1 mm, 2.6 μm) using a 20-min linear gradient at a flow rate of 0.35 mL/min. The column temperature was set at 40°C. Mobile phase buffer A was acetonitrile/water (6/4) with 10 mM ammonium acetate and 0.1% formic acid, whereas buffer B was acetonitrile/isopropanol (1/9) with 10 mM ammonium acetate and 0.1% formic acid. The solvent gradient was set as follows: 30%

B, initial: 30% B for 2 min, 43% B for 5 min, 55% B for 5.1 min, 70% B for 11 min, 99% B for 16 min and 30% B for 18.1 min. Q ExactiveTM HF mass spectrometer was operated in positive (or negative) polarity mode with sheath gas: 20 psi, sweep gas: 1 L/min, auxiliary gas rate: 5 L/min (7 L/min), spray voltage: 3 kV, capillary temperature: 350°C, heater temperature: 400°C, S-Lens RF level: 50, scan range: 114-1700 m/z, automatic gain control target: 1e6, normalized collision energy: 25 eV and 30 eV (20 eV, 24 eV and 28 eV), injection time: 100 ms, isolation window: 1m/z, automatic gain control target (MS2): 1e5, dynamic exclusion: 15 s.

Targeted oxygen lipidomics: LC-MS/MS analyses were performed using an ExionLC™ AD system (SCIEX) coupled with a QTRAP® 6500+ mass spectrometer (SCIEX). Samples were injected onto a C18 Column (10 cm × 2.1 mm) using a 14-min linear gradient at a flow rate of 0.3 mL/min. The eluents were eluent A (0.1% Formic acid) and eluent B (Acetonitrile). The solvent gradient was set as follows: 35% B for 0.5 min, 35-95% B for 9.5 min, 95% B for 10.5 min, 95-35% B for 11 min and 35% B for 14 min. QTRAP® 6500+ mass spectrometer was operated in positive polarity mode with Curtain Gas of 40 psi, Collision Gas of Medium, IonSpray Voltage of -4500V, Temperature: 500°C, Ion Source Gas of 1: 55, Ion Source Gas of 2: 55.

4. Data processing in untargeted lipidomics and targeted oxygen lipidomics

Untargeted lipidomics: The raw data files generated by UHPLC-MS/MS were processed using the Compound Discoverer 3.01 (CD3.1, Thermo Fisher) to perform peak alignment, peak picking and quantitation for each metabolite. The main parameters were set as follows: retention time tolerance for 0.2 minutes, actual mass tolerance for 5 ppm, signal intensity tolerance for 30%, signal/noise ratio for 3 and minimum intensity for 100000. After that, peak intensities were normalized to the total spectral intensity. The normalized data were used to predict the molecular formula based on additive ions, molecular ion peaks and fragment ions. Then, peaks were matched with the Lipidmaps and Lipidblast database to obtain accurate qualitative and relative quantitative results. Statistical analyses were performed using the statistical software R (R version R-3.4.3), Python (Python 2.7.6 version) and CentOS (CentOS release 6.6). When data were not normally distributed, standard transformations were attempted using of area normalization method.

Targeted oxygen lipidomics: Experimental samples were tested using MRM mode (Multiple Reaction Monitoring). The Q3 was used for the metabolite quantification. The Q1, Q3, RT

(retention time), DP (declustering potential) and CE (collision energy) were used for the metabolite identification. The data files generated by HPLC-MS/MS were processed using the SCIEX OS Version 1.4 to integrate and correct the peak. The main parameters were set as follows: minimum peak height for 500, signal/noise ratio for 10 and gaussian smooth width for 3. The area of each peak represents the relative content of the corresponding substance.

Column selection, lipid pretreatment, and detection and result analysis methods refer to the following literature [1-6].

1. Wang, Y.; Armando, A.M.; Quehenberger, O.; Yan, C.; Dennis, E.A. Comprehensive ultra-performance liquid chromatographic separation and mass spectrometric analysis of eicosanoid metabolites in human samples. *J Chromatogr A* **2014**, *1359*, 60-69.
2. Narvaez-Rivas, M.; Zhang, Q.B. Comprehensive untargeted lipidomic analysis using core-shell C30 particle column and high field orbitrap mass spectrometer. *J Chromatogr A* **2016**, *1440*, 123-134, doi:10.1016/j.chroma.2016.02.054.
3. Matyash, V.; Liebisch, G.; Kurzchalia, T.V.; Shevchenko, A.; Schwudke, D. Lipid extraction by methyl-tert-butyl ether for high-throughput lipidomics. *J Lipid Res* **2008**, *49*, 1137-1146, doi:10.1194/jlr.D700041-JLR200.
4. Fahy, E.; Alvarez-Jarreta, J.; Brasher, C.J.; Nguyen, A.; Hawksworth, J.I.; Rodrigues, P.; Meckelmann, S.; Allen, S.M.; O'Donnell, V.B. LipidFinder on LIPID MAPS: peak filtering, MS searching and statistical analysis for lipidomics. *Bioinformatics* **2019**, *35*, 685-687.
5. Kind, T.; Liu, K.H.; Lee, D.Y.; DeFelice, B.; Meissen, J.K.; Fiehn, O. LipidBlast in silico tandem mass spectrometry database for lipid identification. *Nat Methods* **2013**, *10*, 755-+.
6. Zhang, C.L.; Wang, H.L.; Li, P.C.; Hong, C.D.; Chen, A.Q.; Qiu, Y.M.; Zeng, A.P.; Zhou, Y.F.; Hu, B.; Li, Y.N. Mfsd2a overexpression alleviates vascular dysfunction in diabetic retinopathy. *Pharmacological research* **2021**, *171.*
