# Supplementary material for: Bone Marrow Mesenchymal Stem-Cell-Derived Exosomes Ameliorate Deoxynivalenol-Induced Mice Liver Damage
Source: Antioxidants (Basel). 2023 Feb 27;12(3):588. doi: 10.3390/antiox12030588 (PMC10045494; doi:10.3390/antiox12030588)
Supplement: Supplementary file 1 [file antioxidants-12-00588-s001.zip › Supplementary Figures.pdf]

Supplemental Materials

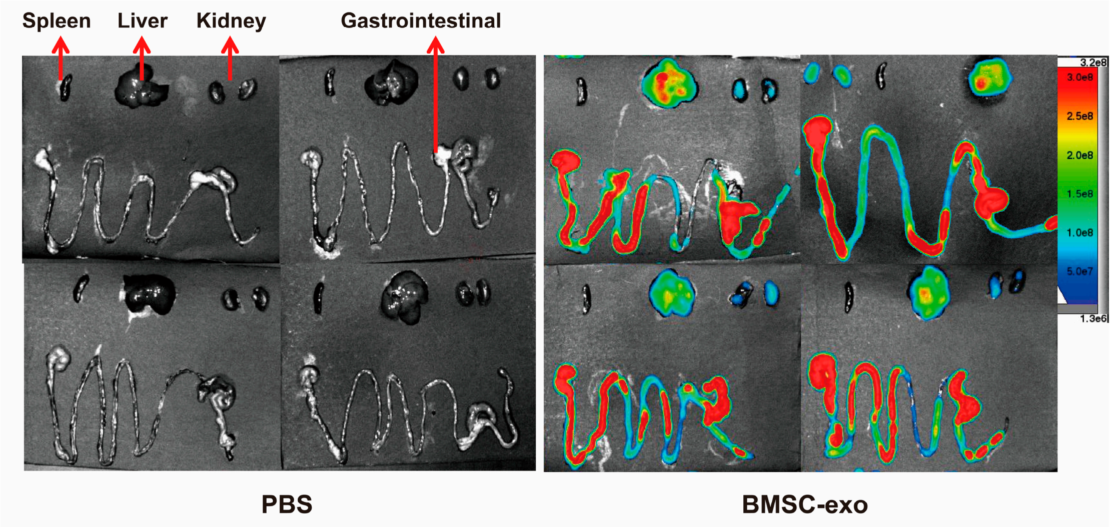

Figure S1 Imaging of ex vivo organs. The organs were removed and imaged ex vivo, and a clear DiR signal was observed in the liver. The fluorescent signal was strongest in the gastrointestinal tract, very low in the kidney, and utterly absent in the spleen.

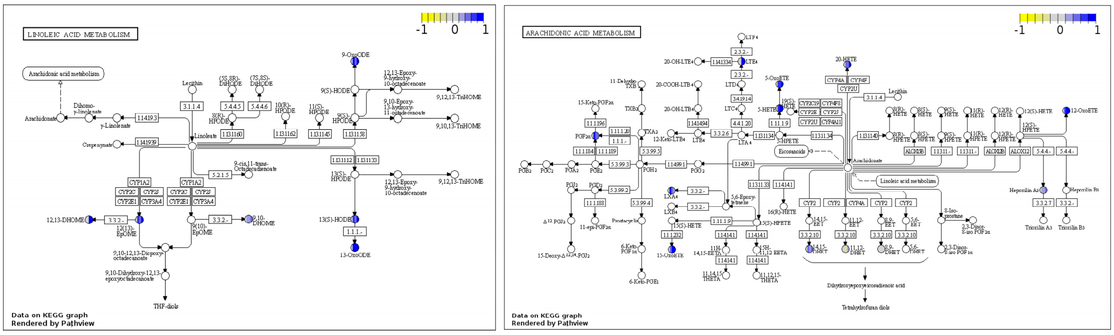

Figure S2 KEGG pathway annotation of detected oxylipins by Pathview.
