# Supplementary material for: Bone Marrow Mesenchymal Stem-Cell-Derived Exosomes Ameliorate Deoxynivalenol-Induced Mice Liver Damage
Source: Antioxidants (Basel). 2023 Feb 27;12(3):588. doi: 10.3390/antiox12030588 (PMC10045494; doi:10.3390/antiox12030588)
Supplement: Supplementary file 1 [file antioxidants-12-00588-s001.zip › Supplementary Table S1.pdf]

Table S1 The VIP value of differential oxylipins between groups

Control and DON	Vip	Control and DON + L-exo	Vip	Control and DON + H-exo	Vip	DON and DON + L-exo	Vip	DON and DON + H-exo	Vip	DON +L-exo and DON + H-	Vip
16-HETE	1.621990456	11-HDoHE	1.569874952	15-oxoETE	1.324163653	14,15-LTC4	2.456739614	12,13-diHOME	1.905730099	4-HDoHE	1.488934443
19-HETE	1.55342997	11-HETE	1.501346447	PGF3a	1.30594183	14,15-LTE4	2.44794053	14,15-LTE4	1.516463391	13-HOTrE	1.443405899
17-HETE	1.523394327	LTE4	1.461927747	12,13-diHOME	1.301331963	LTE4	2.429858661	13-HOTrE	1.498818517	12,13-diHOME	1.408286257
5-oxoETE	1.511127327	17-HETE	1.420071742	19-HETE	1.300471397	dhk PGF2a	2.40498958	12-HEPE	1.483142497	9,10-EpOME	1.399938898
5-HETE	1.503181135	16-HETE	1.418843209	17-HETE	1.285668364	EPA	2.026004609	dhk PGF2a	1.457682587	9-HODE	1.391558425
12-oxoETE	1.486591967	15-oxoETE	1.415327167	9,10-EpOME	1.277700619	AA	1.917791883	14,15-LTC4	1.385352617	18-HEPE	1.388203904
11-HETE	1.459167773	19-HETE	1.400790415	9-HODE	1.271018228	19(20)-EpDPE	1.63182069	LTE4	1.36783947	8-HEPE	1.378424948
PGF3a	1.456188827	12-oxoETE	1.393327112	5-oxoETE	1.257946707	16(17)-EpDPE	1.588202517	15-oxoETE	1.309718469	5-HEPE	1.376702873
15-oxoETE	1.384332219	10-HDoHE	1.373824572	4-HDoHE	1.25789404	6R-LXA4	1.563719274	4-HDoHE	1.304811003	18-HETE	1.371980491
PGF2a	1.276988018	PGF3a	1.353763374	12-oxoETE	1.255598419	PGF2a	1.528749103	11-HETE	1.250037233	11-HEPE	1.342069539
8-HETrE	1.275875386	9-HODE	1.352039265	16-HETE	1.246651905	11,12-diHETrE	1.347241282	18-HEPE	1.215756491	10-HDoHE	1.341981648
5-HETrE	1.274660195	5-HETrE	1.351407707	10-HDoHE	1.239790928	14,15-diHETrE	1.346498184	18-HETE	1.202529396	12,13-EpOME	1.334581789
5-HEPE	1.269188148	5-oxoETE	1.347708124	5-HETrE	1.232457737	9-oxoODE	1.310811756	20-HETE	1.167380987	12-oxoETE	1.325043023
10-HDoHE	1.237610976	9,10-EpOME	1.32585802	5-HETE	1.229390483	HXB3	1.195100114	10-HDoHE	1.137108574	16-HDoHE	1.319863031
13-oxoODE	1.210229977	5-HETE	1.314491481	11-HETE	1.221812665	11-HDoHE	1.154782415	9,10-EpOME	1.135318524	13-HODE	1.312040029
6R-LXA4	1.17922557	17-HDoHE	1.313233336	18-HEPE	1.212427981	13-oxoODE	1.141499809	16-HDoHE	1.123246348	5-oxoETE	1.30674251
11-HDoHE	1.175444941	12,13-diHOME	1.257571859	5-HEPE	1.210072514	12-HEPE	1.085637099	15-HETrE	1.106791002	15-oxoETE	1.292971872
15-HEPE	1.156515394	20-HDoHE	1.243874115	13-oxoODE	1.193057411	12,13-EpOME	1.040215835	9-HODE	1.106011003	8-HDoHE	1.266410606
18-HEPE	1.114081739	8-HDoHE	1.180811766	18-HETE	1.186226748	13-HODE	1.03353018	17-HETE	1.103705028	14-HDoHE	1.258785232
9-HETE	1.104593147	13-oxoODE	1.169406288	13-HOTrE	1.184716793	12,13-diHOME	0.962204593	12-oxoETE	1.089414296	7-HDoHE	1.248540745
20-HDoHE	1.062879717	7-HDoHE	1.165042473	9-HETE	1.170143774	9,10-diHOME	0.853390273	7-HDoHE	1.088627799	11-HETE	1.247952259
9,10-EpOME	1.046108906	16-HDoHE	1.15077681	16-HDoHE	1.150166561	5,15-diHETE	0.835969687	5-HETrE	1.080532446	11-HDoHE	1.219869582
9-HODE	1.029007353	14,15-LTE4	1.144648065	8-HEPE	1.14283472	5-HEPE	0.812142782	5-oxoETE	1.071439064	13-oxoODE	1.212883797
17-HDoHE	1.026073162	4-HDoHE	1.135968071	12,13-EpOME	1.142602149	HXA3	0.786666945	13-HODE	1.071130123	20-HETE	1.210330504
19(20)-EpDPE	1.025500906	9-HETE	1.124715868	15-oxoEDE	1.136692942	8-HEPE	0.758370676	8-HDoHE	1.065785861	15-HEPE	1.207257949
8-HEPE	1.019164468	dhk PGF2a	1.103177497	13-HODE	1.135784972	9-HODE	0.724023446	12,13-EpOME	1.06240449	19-HETE	1.167211185
16(17)-EpDPE	1.00984257	18-HETE	1.080246128	15-HETrE	1.129892406	9,10-EpOME	0.682055868	11-HEPE	1.060148841	20-HDoHE	1.167007699
5S,6R-diHETE	0.992817495	18-HEPE	1.07297709	7-HDoHE	1.124024337	15R-LXA4	0.618683397	15-oxoEDE	1.050701061	17-HDoHE	1.14316792
7-HDoHE	0.992202757	20-HETE	1.032470597	11-HEPE	1.123139454	8-HETrE	0.608516502	9-HETE	1.037021275	9-oxoODE	1.140771959
15R-LXA4	0.99028973	15-HETrE	1.02067455	8-HDoHE	1.121722476	5S,6R-diHETE	0.54771195	8-HEPE	1.020358327	17-HETE	1.121242634
11-HEPE	0.971538961	8-HETrE	1.018852335	20-HETE	1.121158097	15-HEPE	0.532813514	5S,6R-diHETE	0.998263677	15-HETrE	1.057012448
16-HDoHE	0.922013434	14,15-LTC4	1.015977693	11-HDoHE	1.116959282	11-HEPE	0.450054385	19-HETE	0.991447659	5-HETrE	1.051170542
12,13-EpOME	0.921177259	5-HEPE	1.011991384	20-HDoHE	1.112386322	12-HHTrE	0.327715526	5-HEPE	0.987522347	5-HETE	1.048495561
20-HETE	0.898576881	15-oxoEDE	0.975485445	17-HDoHE	1.077654034	13-HOTrE	0.322069109	14-HDoHE	0.985633687	15-oxoEDE	1.047937805
15-oxoEDE	0.896694273	14-HDoHE	0.971113505	15-HEPE	1.076249497	8,15-diHETE	0.31052588	16-HETE	0.982513862	8-HETrE	1.044302727
13-HODE	0.893678722	13-HOTrE	0.899854569	14-HDoHE	1.046538191	11-HETE	0.302469798	6R-LXA4	0.975427176	11,12-diHETrE	1.04340503
9-oxoODE	0.887369428	15R-LXA4	0.898739701	9-oxoODE	1.027258212	10-HDoHE	0.283977622	5-HETE	0.953236436	16-HETE	1.038234399
4-HDoHE	0.88129462	15-HEPE	0.889941361	8-HETrE	0.990349015	PGF3a	0.272781112	11-HDoHE	0.950798331	9-HETE	1.011664321
14,15-diHETrE	0.860343149	11-HEPE	0.835386836	LTE4	0.970346688	5-HETE	0.264203147	17-HDoHE	0.93756554	12-HEPE	0.807758865
12-HHTrE	0.858929558	8-HEPE	0.809409176	15R-LXA4	0.874086755	Adrenic acid	0.248582849	13-oxoODE	0.920313391	12-HHTrE	0.542467219
8-HDoHE	0.842406387	5S,6R-diHETE	0.776802707	5,15-diHETE	0.821935058	19-HETE	0.246044097	PGF2a	0.919400027	Adrenic acid	0.505247485
14-HDoHE	0.803769251	12,13-EpOME	0.728491448	5S,6R-diHETE	0.811816254	8,9-diHETrE	0.217699905	20-HDoHE	0.91682383	6R-LXA4	0.478049341
5,15-diHETE	0.798682131	13-HODE	0.713831375	11,12-diHETrE	0.803177457	18-HEPE	0.21142206	15-HEPE	0.86274913	15R-LXA4	0.442470507
EPA	0.761741441	Adrenic acid	0.688464513	8,15-diHETE	0.791672272	15-oxoETE	0.194329699	8-HETrE	0.824584901	PGF3a	0.418598933
18-HETE	0.724570021	8,15-diHETE	0.679207429	12-HEPE	0.764419919	7-HDoHE	0.178139286	AA	0.774140311	PGF2a	0.379409162
15-HETrE	0.719839171	9-oxoODE	0.590750824	Adrenic acid	0.755324769	18-HETE	0.164659472	PGF3a	0.72462862	8,15-diHETE	0.373301037
LTE4	0.71511608	PGF2a	0.520615891	14,15-LTE4	0.722138565	4-HDoHE	0.140823419	Adrenic acid	0.719615983	LTE4	0.370997898
AA	0.696690054	AA	0.496440247	dhk PGF2a	0.677989994	16-HDoHE	0.133613502	8,15-diHETE	0.719118931	14,15-LTC4	0.295633961
8,15-diHETE	0.678966191	EPA	0.35096114	PGF2a	0.651773406	17-HDoHE	0.130889841	9-oxoODE	0.54068432	8,9-diHETrE	0.2907085
dhk PGF2a	0.593114808	12-HHTrE	0.339998599	8,9-diHETrE	0.574618946	5-oxoETE	0.125354379	EPA	0.521579393	DHA	0.274564809
HXB3	0.579981476	5,15-diHETE	0.270242799	14,15-LTC4	0.529007303	DHA	0.103279723	5,15-diHETE	0.514014836	5S,6R-diHETE	0.239381866
8,9-diHETrE	0.554595443	11,12-diHETrE	0.21372446	DHA	0.495490962	5-HETrE	0.100339074	16(17)-EpDPE	0.481136483	16(17)-EpDPE	0.238509604
HXA3	0.475235946	12-HEPE	0.199757697	9,10-diHOME	0.431415128	8-HDoHE	0.08431306	19(20)-EpDPE	0.436872991	14,15-LTE4	0.216203469
9,10-diHOME	0.457159952	14,15-diHETrE	0.173320438	19(20)-EpDPE	0.424899711	9-HETE	0.08076318	11,12-diHETrE	0.431648507	11,12-diHETrE	0.206527064
DHA	0.354866876	9,10-diHOME	0.167434007	14,15-diHETrE	0.359107523	15-HETrE	0.066596191	9,10-diHOME	0.353526513	dhk PGF2a	0.204474142
14,15-LTC4	0.323127972	HXA3	0.155994194	6R-LXA4	0.324530601	16-HETE	0.066340817	15R-LXA4	0.323064303	EPA	0.161558041
12,13-diHOME	0.322094632	DHA	0.134390916	HXA3	0.261734497	20-HDoHE	0.062674417	HXA3	0.27123351	5,15-diHETE	0.102704564
14,15-LTE4	0.305393008	19(20)-EpDPE	0.117802662	AA	0.254742115	15-oxoEDE	0.055853249	14,15-diHETrE	0.22145537	AA	0.063578768
12-HEPE	0.298985099	8,9-diHETrE	0.115588115	16(17)-EpDPE	0.23725756	14-HDoHE	0.035408758	DHA	0.156156231	9,10-diHOME	0.027245997
11,12-diHETrE	0.266643159	6R-LXA4	0.054332909	HXB3	0.22893315	17-HETE	0.034261842	HXB3	0.104192766	14,15-diHETrE	0.020947081
13-HOTrE	0.264419789	16(17)-EpDPE	0.023188757	EPA	0.130626131	20-HETE	0.012393274	8,9-diHETrE	0.100789779	19(20)-EpDPE	0.014675309
Adrenic acid	0.008056823	HXB3	0.019643966	12-HHTrE	0.066426584	12-oxoETE	0.001515428	12-HHTrE	0.016708737	HXB3	0.009229036
